# Comparative Genomics Analysis in Grass Species Reveals Two Distinct Evolutionary Strategies Adopted by R Genes

**DOI:** 10.1038/s41598-019-47121-8

**Published:** 2019-07-24

**Authors:** Yinan Zhang, Meijun Guo, Jie Shen, Xie Song, Shuqi Dong, Yinyuan Wen, Xiangyang Yuan, Pingyi Guo

**Affiliations:** Agronomy College, Shanxi Agricultural University, Taigu, 030801 China

**Keywords:** Plant evolution, Population genetics

## Abstract

Resistance genes play an important role in the defense of plants against the invasion of pathogens. In *Setaria italica* and closely related grass species, R genes have been identified through genetic mapping and genome-wide homologous/domain searching. However, there has been to date no systematic analysis of the evolutionary features of R genes across all sequenced grass genomes. Here, we determined and comprehensively compared R genes in all 12 assembled grass genomes and an outgroup species (*Arabidopsis thaliana*) through synteny and selection analyses of multiple genomes. We found that the two groups of nucleotide binding site (NBS) domains containing R genes—R tandem duplications (TD) and R singletons—adopted different strategies and showed different features in their evolution. Based on *K*_a_/*K*_s_ analysis between syntenic R loci pairs of TDs or singletons, we conclude that R singletons are under stronger purifying selection to be conserved among different grass species than R TDs, while R genes located at TD arrays have evolved much faster through diversifying selection. Furthermore, using the variome datasets of *S*. *italica* populations, we scanned for selection signals on genes and observed that a part of R singleton genes have been under purifying selection in populations of *S*. *italica*, which is consistent with the pattern observed in syntenic R singletons among different grass species. Additionally, we checked the synteny relationships of reported R genes in grass species and found that the functionally mapped R genes for novel resistance traits are prone to appear in TDs and are heavily divergent from their syntenic orthologs in other grass species, such the black streak R gene *Rxo1* in *Z*. *mays* and the blast R gene *Pi37* in *O*. *sativa*. These findings indicate that the R genes from TDs adopted tandem duplications to evolve faster and accumulate more mutations to facilitate functional innovation to cope with variable threats from a fluctuating environment, while R singletons provide a way for R genes to maintain sequence stability and retain conservation of function.

## Introduction

Resistance (R) genes confer to plants innate immunity against a broad spectrum of pathogens, including viruses, bacteria, fungi and nematodes, through expressing matching avirulence genes in a “gene-for-gene” manner^1–5^. There are five main recognized groups of R proteins based on differences in the domains of cloned R genes^1,6–10^. Among these, the largest group encodes receptor-like proteins that feature a conserved nucleotide-binding site (NBS) domain accompanied by a C-terminal leucine-rich repeat (LRR) domain that can recognize antigen proteins of a pathogen. The NBS R genes are further separated by an N-terminal Toll/Interleukin-1 Receptor (TIR domain) or a coiled-coil (CC motif) homology region^11–13^. The TIR domain is the component of a large R gene subgroup in dicot, conifer, and moss genomes, but the domain is almost nonexistent in monocots^8,12,14,15^.

Whole-genome duplication (WGD) and tandem duplication (TD) contributed to the expansion of the largest NBS R gene group in plant genomes^16^. The NBS R genes have been studied via whole-genome sequencing in dicot and monocot species, including *Arabidopsis thaliana*, *Oryza sativa*, *Populus tremula*, *Carica papaya*, *Medicago truncatula* and *Vitis vinifera*^7–9,17–19^. According to previous studies, approximately 0.2–2% of the genes in plant genomes are NBS R genes, with papaya—which has not experienced recent whole genome duplication—retaining relatively fewer NBS R genes in its genome^17^. In the genomes of *A*. *thaliana*, *O*. *sativa*, and *C*. *papaya*, the majority of the NBS R genes are clustered in local regions, which is consistent with the consequences of tandem duplication events^7–9,16,17,20–22^.

R genes play an important role in monocots, including field crop species. Previous studies have mapped and cloned many R genes involved in disease resistance and have found that some R genes were under strong selection related to pathogen *Avr* genes^23^. This work has contributed to the breeding of disease-resistant crops in agricultural production. Among the more well-studied R genes, the majority are NBS R genes. In *Zea mays*, *Rxo1* has been cloned as a resistance gene against bacterial streak disease^24^. *Rxo1* is an NBS R gene that confers resistance to a set of pathogen strains, including an unrelated pathogen that causes bacterial stripe in *Sorghum bicolor* and *Z*. *mays*. *Rxo1* retained its resistance function when used as a transgene into *O*. *sativa* as a defense against the bacterial streak disease, demonstrating the shared functional mechanism and the feasibility of cross-species utility of R genes among grass species. In *O*. *sativa*, more than 20 NBS R genes have been cloned to confer resistance against the blast and bacterial blight diseases. Fei *et al*.^25^, Zeng *et al*.^26^, and Wu *et al*.^27^ independently cloned the blast resistance gene *Pia* from the loci of NBS R gene TD arrays by genetic mapping of resistant cultivars of *O*. *sativa*. Guo *et al*. cloned several *O*. *sativa* blast resistance genes through paralogous comparison based on sequence homology^28^. Furthermore, Yang and co-authors analyzed rapidly evolving R gene families in maize, sorghum, brachypodium, and rice, and found that after transforming some of these genes into rice, the transformed plants gained blast disease resistance^29^. This suggested that fast evolving R genes play an important role in functional innovations of resistance. Though many NBS R genes have been determined to maintain resistant traits, there are still a large number that have not been functionally characterized and thus are a rich resource for resistance function innovation, mechanism investigation, and potential breeding utilization.

Systematic determination and comparison of R genes among multiple closely related species will help to reveal evolutionary features with regard to expansion, divergence, and function innovation. R genes have been studied in many grass species, including *O*. *sativa*, *Z*. *mays*, and *Setaria italica*^9,13,22,30^ following whole-genome sequencing of these species, and several studies have been performed with limited comparisons among 3–4 species^31,32^. However, genome-wide determination and comparison of R genes across all sequenced grass species has not yet been done. In this study, we focused on all grass species whose genomes have been sequenced and released (11 genomes), together with two outgroup species. We performed comprehensive comparisons of R genes after genome-wide determination under the same rules in these 13 species. We found that R gene loci showed high levels of conservation among species, and R genes from TD and singleton loci adopted different strategies in the functional evolution of disease resistance.

## Results

### Identification of R genes in *Setaria italica* and closely related grass species

**Figure 1:**
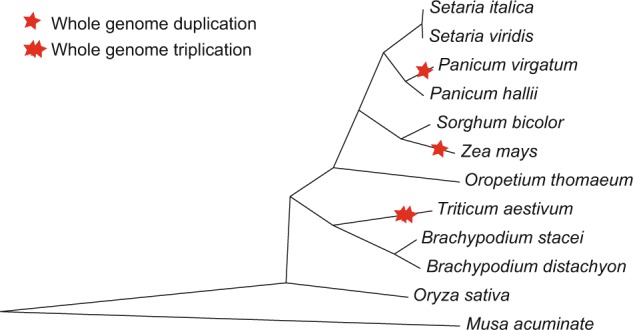
Phylogenetic tree built on *K*_s_ loci of syntenic orthologous genes among the 12 grass species. Red stars denote whole-genome duplication events; double red stars denotes whole genome triplication events.

Genome sequences and predicted gene information for 13 species (Table 1) were downloaded from the databases phytozome and PlantGDB for R genes analysis. To check their phylogeny relationships accurately—as a guide for analyzing R gene evolution—we performed pairwise genomic synteny analysis between *S*. *italica* and each of the other species (Methods). Using the genome of *S*. *italica* as a control, *P*. *virgatum* and *Z*. *mays* were confirmed as tetraploids; *T*. *aestivum* is hexaploid, while the other species are diploids (the distant outgroup species *M*. *acuminate* and *A*. *thaliana* are not applicable) (Supplementary Figs S1–S9). Based on the syntenic gene datasets, we obtained 357 groups of complete syntenic orthologs (syntenic orthologs exist in all studies genomes) among the 12 grass species (except for the dicot *A*. *thaliana*). We further extracted 10,443 *K*_s_ loci (coding variants that would not cause amino acids changes to the corresponding proteins) from these 357 syntenic ortholog groups. These loci were then concatenated and used to build the phylogenetic tree under the neighbor-joining algorithm in MEGA. As shown in Fig. 1, with *M*. *acuminate* as the outgroup, the evolutionary relationships of these 12 grass species were clear, meaning that the phylogeny serves as a good reference for evolutionary analysis of R genes among these species.

**Table 1:** Genomic information of 12 grass species and one outgroup species studied in this work.

Index	Species	#Chr*	#Genes	Genome (Mb)	WGP**
1	*Setaria italica*	9	34584	405.74	1
2	*Setaria viridis*	9	35214	394.9	1
3	*Panicum virgatum*	18	91838	1689.57	2
4	*Panicum hallii*	9	37232	554.13	1
5	*Sorghum bicolor*	10	34211	732.15	1
6	*Zea mays*	10	88760	2067.86	2
7	*Oropetium thomaeum*	/	28437	243.17	1
8	*Triticum aestivum*	21	107891	14547.26	3
9	*Brachypodium stacei*	10	29898	234.14	1
10	*Brachypodium distachyon*	5	34310	271.16	1
11	*Oryza sativa*	12	42006	374.47	1
12	*Musa acuminate*	11	36528	472.96	—
13	*Arabidopsis thaliana*	5	27751	119.67	—

^*^Chr: Chromosomes; **WGP: whole genome polyploidization.

**Figure 2:**
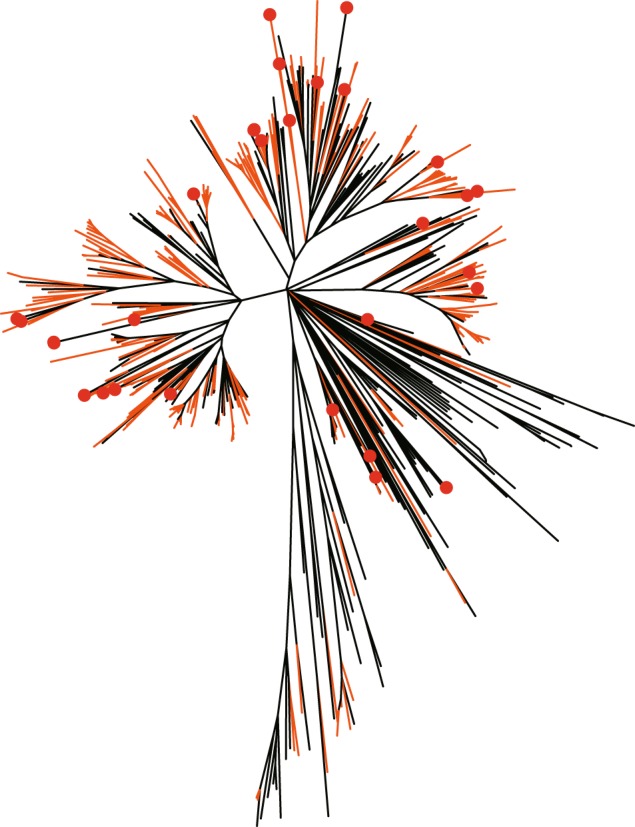
The phylogenetic tree of 535 R genes in *S*. *italica*. The tree was built with the maximum-likelihood algorithm by MEGA software. The red full circles denote *S*. *italica* specific R genes, i.e., *S*. *italica* R genes that have no syntenic orthologs in any of the other 11 grass species. Branches colored in orange are R genes from tandem duplication arrays, others are R gene singletons.

Domain compositions of well-determined R genes in model species *A*. *thaliana* and *O*. *sativa* were used to search for R genes in those 13 genomes. A total of 202 and 456 R genes of *A*. *thaliana* and *O*. *sativa*, respectively, were collected from previous studies^8,22^. First PfamScan^33^ was used to search for domains in these R genes. In all, 55 domains were identified from R genes of the two model plant species (Supplementary Table S1). These domains were further used to determine candidate R genes in the genomes of all 13 species using the tool hmmsearch^34^. After that, we obtained 1,264 R gene candidates in *S*. *italica*. By filtering genes without the NBS domain, we finally obtained 535 NBS R genes (referred as R genes hereafter) in *S*. *italica*. Figure 2 shows the phylogenetic relationships of these 535 R genes determined in *S*. *italica* using the maximum likelihood algorithm in MEGA. With the same method, we determined a set of R genes ranged from 97–2,747 copies (accounting for 0.34–2.55% of the total number of genes) in these 13 genomes (Table 2). The hexaploid *T*. *aestivum* had the most R genes (2,747), while the tetraploid *P*. *virgatum* had the second most R genes (1,267). In the diploids, *O*. *sativa* (587) and *S*. *italica* (535) had the most R gene copies, while *O*. *thomaeum* had the least number of copies (97). The R gene copy numbers show association to the polyploidization levels of the corresponding species, except for the tetraploid *Z*. *mays*, which has relatively fewer (306) R genes. When comparing numbers of R genes to total genes, *T*. *aestivum* had the highest ratio of R genes (2.55%), while *O*. *thomaeum* had the smallest ratio (0.34%), followed by *Z*. *mays* (0.35%), *M*. *acuminate* (0.52%), and *A*. *thaliana* (0.83%). R genes can be further classified into three main groups as NBS, CC-NBS, and TIR-NBS. The 535 R genes in *S*. *italica* can be further separated into 14 subgroups on the basis of composition and order of domains (Table 3). Among these R genes, the NBS group R genes had the most copies, while the TIR-NBS group R genes had the least number of copies. The other genomes shared similar distribution patterns of R gene subgroups, except for *A*. *thaliana*, in which TIR domain-containing R genes had the most copies (Supplementary Table S2). In contrast to *A*. *thaliana*, the 12 grass species had very limited numbers of copies of TIR-containing R genes, which is consistent with previous reports^8,12,14,15^.

**Table 2:** Statistics summary of R genes in the 13 studied species.

Index	Species	#R candidate	#NBS R	NBS R/Gene	#R TD*	#TD R**	TD R/NBS	#R singleton	#R locus
1	*S*. *italica*	1,264	535	1.55%	106	303	0.57	232	338
2	*S*. *viridis*	1,152	465	1.32%	92	241	0.52	224	316
3	*P*. *virgatum*+	2,949	1,267	1.38%	212	524	0.41	743	955
4	*P*. *hallii*	1,101	420	1.13%	86	234	0.56	186	272
5	*S*. *bicolor*	1,036	425	1.24%	82	233	0.55	192	274
6	*Z*. *mays+*	1,101	306	0.34%	36	79	0.26	227	263
7	*O*. *thomaeum*	410	97	0.34%	4	8	0.08	89	93
8	*T*. *aestivum++*	5,712	2,747	2.55%	547	1,588	0.58	1,159	1,706
9	*B*. *stacei*	945	377	1.26%	78	203	0.54	174	252
10	*B*. *distachyon*	1,091	464	1.35%	105	267	0.58	197	302
11	*O*. *sativa*	1,390	587	1.40%	116	339	0.58	248	364
12	*M*. *acuminate*	863	190	0.52%	24	67	0.35	123	147
13	*A*. *thaliana*	896	229	0.83%	49	124	0.54	105	154

*R TD means tandem duplication array that is composed of R genes;

**TD R means the R genes comes from a tandem array; +: tetrapolyploidization; ++: hexapolyploidization.

**Table 3:** The subgroups of 535 R genes in *S*. *italica*.

Index	Domains	#Copy	Sum
1	CC-NBS-LRR	159	230
2	CC-NBS	41	
3	CC-NBS-NBS-LRR	14	
4	CC-NBS-NBS	12	
5	CC-NBS-LRR-LRR	3	
6	CC-LRR-NBS-LRR	1	
7	NBS-LRR	156	302
8	NBS	88	
9	NBS-NBS	45	
10	NBS-NBS-LRR	10	
11	NBS-LRR-LRR	2	
12	LRR-NBS-NBS-LRR	1	
13	TIR-NBS	2	3
14	TIR-NBS-TIR	1	
Total		535	

### High level of synteny relationships of R loci among grass species

There are two groups of R genes based on whether or not they are distributed continuously or occur singly. The closely distributed R genes are called tandem R genes (R genes from TD arrays), while the others are called R gene singletons. In order to separate the two groups of R genes, we considered continuously distributed R genes that were interrupted by less than five non-R genes as R genes in TDs (referred as R TDs hereafter) (Methods). In all, we determined 106 R TDs (comprising 303 R genes) in *S*. *italica*, accounting for 56.63% of a total of 535 NBS R genes (Table 2). The remaining 232 R genes are singletons. These R TD genes are colored in orange in Fig. 2. As shown in Fig. 2, the R TD and singleton genes are generally mixed within the phylogeny, with more R singletons clustered in two more divergent branches (bottom-right part of Fig. 2). This indicates that these R singletons were formed much earlier and were conserved as singletons for a longer time than R TDs in the upper branches of the tree. Using the same method, we obtained 4–547 R TDs (comprised of 8–1,588 R genes) in the other genomes, accounting for 3.72–57.75% of the total number of R genes in corresponding genomes (Table 2). We further selected one of the R genes in each of the 106 TDs in *S*. *italica* as a representative gene of each R TD. Together with the 232 R singletons, there were 338 R gene loci (referred as R loci hereafter) in the genome of *S*. *italica*. Similarly, we determined 93–1,706 R loci in the other genomes (Table 2). Additionally, we investigated the domain-related subgroups of R genes in R TDs, and found that different R domain subgroups showed no significant distribution differences in relation to R TDs. However, for the subgroups of R genes that had less than five copies, we found that they had a high probability to be from R TDs. These R genes with limited copies show duplicated or abnormal orders of domains. It seems reasonable that those genes are R genes from TDs that originated from domain rearrangement of duplicated R genes in the same TDs.

Most R genes in *S*. *italica* had syntenic orthologs in the other grass genomes. Based on whole-genome synteny analysis (see Methods), we obtained syntenic orthologous gene sets between each pair of the 13 species, i.e., 78 pairwise comparisons in total (Supplementary Table S3). Based on the datasets, we checked the syntenic orthologs of the 338 *S*. *italica* R loci in other genomes. We found that 315 out of 338 R loci (93.20%) had syntenic orthologous loci in at least one of the other genomes, and 131 R loci (38.76%) had syntenic orthologs in more than half (6) of all compared genomes (Supplementary Fig. S10). We further analyzed the synteny relationships of R loci for the two R gene groups separately. For these 106 R TDs in *S*. *italica*, we found that 102 (96.23%) had syntenic orthologous loci in at least one of the other species, among which 94 loci were R TDs in at least one of the other species; for the 232 R singleton loci, we found that 213 (91.81%) had syntenic orthologs in other species, among which 98 syntenic loci were R TDs in at least one species. These four R TDs and 19 R singletons that are specific to *S*. *italica* are denoted by red circles in Fig. 2. There were no clear clustering differences to other R genes in *S*. *italica*, indicating that they originated from transposition duplications of these R genes that have synteny relationships to other genomes.

### R genes evolved rapidly through tandem duplications and are conserved in singletons

Most of the R loci retain syntenic relationships to R loci in the other grass genomes. After removing the two distant outgroups *M*. *acuminate* and *A*. *thaliana*, which have limited syntenic orthologs to the 11 grass genomes, as well as *T*. *asteria* whose genome was extremely expanded, we obtained syntenic orthologous gene families among the ten grass species based on the aforementioned pairwise syntenic gene datasets. In all, we obtained 112,723 such syntenic gene families among the ten genomes. We found that 785 out of the 112,723 syntenic orthologous families belonged to the R loci syntenic gene families. Among these, 359 syntenic families of R loci had syntenic orthologs in two or more species, while the other 426 R loci families were unique in only one of the ten grass genomes (17–143 R loci families were species-specific to one of the 10 genomes) (Supplementary Table S4). For the 359 syntenic families, 168 families contained R TDs in at least one of these syntenic R loci (46.90%). For the 426 species-specific R loci, 85 were R TDs (19.95%), which is significantly lower than that of shared R loci among species (χ^2^ test, *P* = 1.63 × 10^–8^). This indicates that the shared R loci tended to retain more R TD sequences in the evolutionary expansion of R genes, while the species-specific R genes tended to be retained in fewer copies as R singletons.

**Figure 3:**
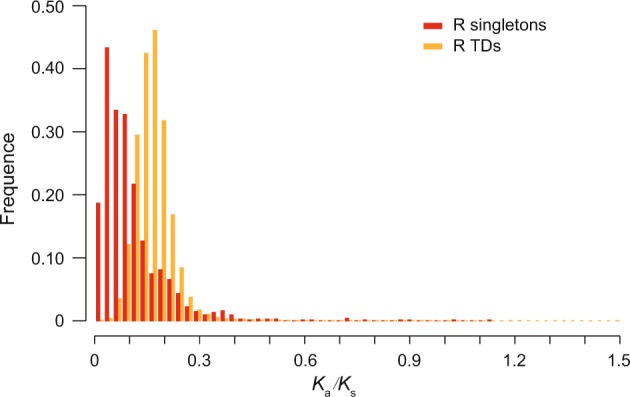
The frequency distribution of *K*_a_/*K*_s_ values between syntenic R gene pairs from R singletons (colored in red) or R TD arrays (colored in light orange).

R genes in TDs apparently evolve more rapidly than R singletons. We calculated *K*_a_ (the rate of non-synonymous mutations per non-synonymous locus) and *K*_s_ (the rate of synonymous mutations per synonymous locus) values between pairs of genes from the R syntenic orthologous families among the ten grass species in order to estimate the selection strength imposed on syntenic R genes. We further investigated the frequency distributions of these *K*_a_, *K*_s_ and *K*_a_*/K*_s_ values for R genes from TDs and singletons separately. For R TDs, we have 39,855 such pairwise syntenic orthologs comparisons, while for R singletons, we obtained 1,598 pairwise comparisons. The *K*_a_ values from R gene singletons were much less than those of R genes from TDs (Supplementary Fig. S11). This suggests that R genes in TDs have been evolving and diverging much faster than R gene singletons. We further examined the differences of *K*_a_/*K*_s_ values within the same comparisons; the analysis showed that R gene singletons have smaller *K*_a_/*K*_s_ values than those of R genes from TDs (Fig. 3). The result indicates that the R gene singletons are under much stronger purifying selection than R TDs genes. These findings together suggest that R genes have expanded and rapidly evolved through TDs, while R genes with important original or newly evolved functions would be conserved as or reduced into singletons.

### R genes are under selection in populations of *S*. *italica*

R genes have accumulated more mutations than other genes. From the whole-genome resequencing data of 916 *S*. *italica* accessions reported and released previously^35^, we determined 2,156,941 SNPs and 189,440 InDels variants from the *S*. *italica* population after mapping the resequencing data to the reference genome of *S*. *italica*^36^ (See Methods). We then annotated these variants (detailed information is listed in Supplementary Tables S5 and S6). There were 704,436 SNPs and 89,804 InDels located in the genic regions of 34,584 genes in *S*. *italica*. We further considered SNPs that caused non-synonymous mutations or splicing alterations (within two bps of splicing loci) as well as InDels located at coding sequences or splicing loci as functional variants, as they lead to changes in the final protein sequences. There were 79,296 such functional variants obtained (70,671 SNPs and 8,625 InDels), an average of 2.29 variants per gene. We then examined the 535 R genes and found that they had accumulated 9,006 functional mutations (8,466 SNPs and 540 InDels), which is approximately16.83 functional variants per R gene. This was significantly higher than the average level of all genes (2.29 functional variants per gene) (χ^2^ test, *P* < 2.20 × 10^−16^) (Table 4). Furthermore, we checked the two aforementioned R gene groups and found that the R genes from R TDs accumulated 20.70 functional mutations per gene, while these singletons only had 11.78 such variants per gene, significantly lower than that of R TDs (χ^2^ test, *P* = 3.68 × 10^−10^). This suggests that R genes in tandem arrays have evolved more rapidly than R gene singletons.

**Table 4:** Functional mutation comparison between total genes and R genes.

	#Genes	#Functional mutations	Functional mutations/Genes	P values (χ2 test)
Total gene	34,584	79,296	2.29	<2.20 × 10^−16^
NBS R gene	535	9,006	16.83	
*R singleton*	232	2,734	11.78	3.68 × 10^−10^
*R TD*	303	6,272	20.70	

**Figure 4:**
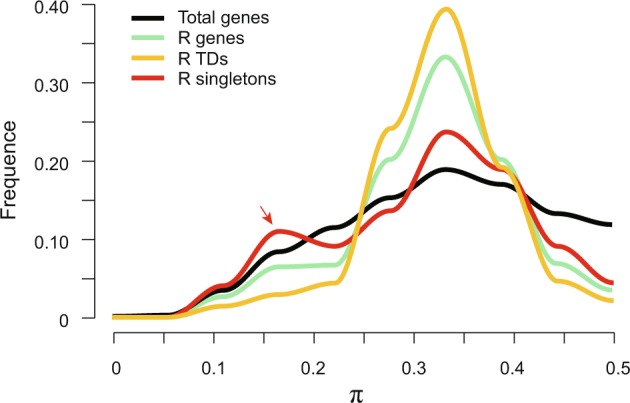
The distribution of π values of total genes (colored in black) and R genes (light blue) in the population of *S*. *italica*. R genes were further separated into R singletons (red) and R tandem arrays (light orange).

R genes are under stronger selection than other genes. Using the variome datasets from *S*. *italica* populations, we evaluated the selection pressure imposed on genes by calculating the measures π^37^ and Tajima’s D^38^. The values of π range from 0 to 1 and estimate the level of polymorphism of given sequences in a population, while Tajima’s D estimates the degree of purifying or balancing selection. We obtained 34,584 values of π and Tajima’s D corresponding to 34,584 genes in the genome of *S*. *italica*. Figs 4 and S12 show the distributions of these values for all genes and R genes, respectively. The R genes are distributed as a steep peak around the π value of 0.35, which suggests that they are under more divergent selection compared to all genes in the population of *S*. *italica*. As mentioned above, we estimated the selection strength for groups of R TDs and R singletons separately and found that R singletons exhibited distribution peaks at lesser values of π and Tajima’s D (Figs 4 and S12). This suggests that the R genes from TDs are under balancing selection, while areas of R singleton genes are under purifying selection. Taken together, the results suggest that R genes have undergone tandem duplications that promoted rapid evolution, while singletons enabled R genes to retain and conserve sequence and function.

### Newly mapped R genes show strong divergence among species

Syntenic orthologs of newly mapped functional R genes are under divergent selection among grass species. *Pi37* is an R gene that confers the trait of blast disease resistance in rice^25^. We examined the synteny orthologs of *Pi37* in the 11 grass genomes and found that ten of the 11 species had syntenic R gene loci to *Pi37* except *Z*. *mays* (Table 5), with two loci in *S*. *italica* and *O*. *thomaeum* being involved in micro-translocations in local genomic regions. Although these syntenic orthologs were inherited from a common locus of a most recent common ancestral genome, their coding sequences have heavily diverged (identity: 27.54–68.34%, coverage: 38.84–100%; Table 5). This indicates that these syntenic R loci have rapidly evolved and diversified in different species, and that they may have evolved different resistance functions. The previously reported blast resistance gene *Pi37* is located in an R TD (syntenic loci in *P*. *hallii* and T. aestivum also are R TDs) in *O*. *sativa*. This R TD locus may have originated after the divergence of these species, and the R TD then contributed to blast resistance functional innovation in *O*. *sativa*. The syntenic gene of *Pi37* in *S*. *italica* is *Seita*.*5G337400*.*1*, which is an R singleton and is involved in a local translocation. *Seita*.*5G337400*.*1* showed the lowest identity to *Pi37* of *O*. *sativa*, and thus it may not have the same blast resistance function as that of *O*. *sativa Pi37*. Meanwhile, *Seita*.*5G337400*.*1* has been under selection in populations of *S*. *italica* as indicated by π (0.18) and Tajima’s D (0.20) compared to other genes (Figs 4 and S12). However, the target of selection on *Seita*.*5G337400*.*1* in *S*. *italic* populations should be different to that of blast resistance of *Pi37* in *O*. *sativa*.

**Table 5:** The syntenic orthologous genes of *O*. *sativa* blast disease resistance gene *Pi37* in the other 11 grass species.

Species	Pi37 synteny orthologs	Identity%	Coverage%	E-value	Tandem array
*O*. *sativa*	*Os01g57310*.*1* (*Pi37*)	/	/	/	*Os01g57270*.*1*; *Os01g57280*.*1*; *Os01g57310*.*1*; *Os01g57340*.*1*
*B*. *distachyon*	*Bradi2g51807*.*3*	68.34	100	0	/
*B*. *stacei*	*Brast01G124500*.*1*	68.03	100	0	/
*T*. *aestivum*	*TraesCS3A02G316800*.*1*	66.26	56.90	0	*TraesCS3A02G316800*.*1*; *TraesCS3A02G316900*.*1*
	*TraesCS3B02G349500*.*1*	66.29	47.60	0	*TraesCS3B02G349500*.*1*; *TraesCS3B02G349600*.*2*; *TraesCS3B02G349800*.*1*
	*TraesCS3D02G314300*.*4*	64.56	55.12	0	*TraesCS3D02G314300*.*4*; *TraesCS3D02G314400*.*1*
*O*. *thomaeum*	*Oropetium_20150105_12744A*	45.11	38.84	4.42E-84	/
*Z*. *mays*	/	/	/	/	/
*S*. *bicolor*	*Sobic*.*003G317300*.*2*	62.11	100	0	/
*P*. *hallii*	*Pahal*.*E01385*.*1*	61.95	100	0	*Pahal*.*E01385*.*1*; *Pahal*.*E01390*.*1*
*P*. *virgatum*	*Pavir*.*5KG572900*.*1*	61.53	92.48	0	/
*S*. *viridis*	*Sevir*.*5G344900*.*1*	55.43	100	0	/
*S*. *italica*	*Seita*.*5G337400*.*1*	27.54	48.14	1.68E-40	/

We further analyzed the syntenic orthologous genes of *Z*. *mays Rxo1* in the 11 grass species. *Rxo1* is an R gene that has been mapped and reported to confer resistance against the bacterial streak disease^24^. There were seven species that retained such syntenic loci (Supplementary Table S7), and all are involved in local micro-translocations. We found that all of these syntenic loci were R singletons, except for *Z*. *mays Rxo1* located in an R TD. The loci show even poorer sequence similarity (identity: 26.55–27.70%, coverage: 38.84–100%) than the *Pi37* syntenic orthologs, indicating greater divergence among these syntenic orthologous genes of *Rxo1*. The syntenic gene of *Rxo1* in *S*. *italica* is *Seita*.*6G072000*.*1*, which has been under diversifying selection in *S*. *italica* (π: 0.38 and Tajima’s D: 1.48). These findings for *Pi37* and *Rox1* supported the suggestion that R TDs contributed to the functional innovation of R genes in different species to cope with distinct environmental threats, and thus it is difficult to predict the resistance functions of R genes based on synteny relationships to genes with known defense traits in other species.

## Discussion

R genes from TD arrays are under more rapid evolution than R gene singletons. Whole genome synteny comparisons of R gene loci found that most R genes have syntenic orthologous R loci in other species. This suggests that R gene loci are relatively conserved among species, and R genes are mostly increased through TDs rather than by transposition or segmental duplications. These R gene TD arrays then evolved new functions against disease invasions. R genes featured in novel functions continuously evolve to adapt to changing environments and biotic disease challenges, so it is reasonable that R genes have evolved faster than other genes. However, when comparing the two groups of R genes (R TDs and singletons), we further found that syntenic families of R genes from TD arrays had higher *K*_a_ values among genomes than R gene singletons. This suggests that R genes from TD arrays evolved faster than R singletons, and that TD arrays act as an important incubators for R genes to evolve temporary and variable functions to cope with novel disease factors. This conclusion is supported by the fact that many newly-mapped R genes are distributed in TD arrays, for example the blast resistance R gene *Pi37* of *O*. *sativa* and the black streak disease resistance R gene *Rox1* in *Z*. *mays*^24^. It is also consistent with previous findings that rapidly evolving R gene families contribute to the functional innovation of blast disease resistance after transforming them into rice^29^. *K*_a_/*K*_s_ values further supported the conclusion that compared to R TD arrays, R singletons are under selection to be conserved. This indicates that when an important resistant-like function (long lasting, basic, or broad spectrum effect) has evolved, selection will retain the new variant in singleton status to prevent it from further sequence variation. These results support the hypothesis that the R genes from TD arrays and singletons adopt different strategies and play different roles in R gene evolution.

R genes from TD arrays are apparently under diversifying selection in *S*. *italica*. The variome dataset generated from the resequencing of 916 accessions of *S*. *italica*^35^ serves as a valuable resource for examining the features of genetic polymorphism in the *S*. *italica* population. By comparing the SNP and InDel variants between R genes and others, we found that R genes accumulated more mutations than other genes, which suggests that R genes evolved more rapidly than other genes. Furthermore, when taking these functional mutations into account, we found that R genes had significantly more such variants than non-R genes. Comparisons of R genes from TD arrays and singletons showed that R TDs had the highest average number of functional mutations per gene, suggesting that R genes from TD arrays are among the most changeable gene groups. Moreover, the differences in selection pressure imposed on R TD arrays, R singletons, and non-R genes resulted in R gene TDs having the highest level of polymorphism. For R singletons, there is selection for low levels of polymorphism, i.e., purifying selection. Considering that low-depth resequencing data cannot determine structural mutations, R genes may involve gene copy variants, despite SNP and InDel mutations. The current data may not reveal the full variation information of R genes in *S*. *italica*, but the data do reflect the general feature patterns.

R genes are one of the most important gene families in plants, as they evolve relatively rapidly and adopt different strategies during the process of accumulating mutations and promoting functional innovations. In this work, we systematically examined the R genes in grass genomes and compared them across different species as well as in the population of *S*. *italica* using comparative genomics and population genomic analysis, respectively. We found that R gene loci show conservation among grass species, and that tandem duplication is the major pathway for R gene expansion in diploid genomes. More importantly, we found that R TD arrays and R singletons differed in their evolutionary strategy, with R TD arrays promoting functional innovations to cope with new disease threats, while R singletons were more likely to maintain and conserve important resistance functions over evolutionary time.

## Materials and Methods

### Resources of genome and gene sequences

The 13 genome datasets (genome sequences, gene prediction coordinates, gene coding sequences and peptides) were downloaded from the databases phytozome (https://phytozome.jgi.doe.gov/pz/portal.html) and PlantGDB (http://www.plantgdb.org/). The 11 grass species were: *Triticum aestivum*, *Brachypodium distachyon*, *Brachypodium stacei*, *Oryza sativa*, *Oropetium thomaeum*, *Zea mays*, *Sorghum bicolor*, *Panicum virgatum*, *Panicum hallii*, *Setaria italica*, *Setaria viridis*, and two species as outgroups, *Musa acuminate* and *Arabidopsis thaliana*. Resequencing data of 916 *S*. *italica* accessions were downloaded from the European Nucleotide Archive (http://www.ebi.ac.uk/ena/) with the accession number ERP002070^35^.

### Determination of R genes

Four main steps were applied to identify R genes in the 13 species. First, we retrieved the gene and protein sequences of 202 R genes from *A*. *thaliana*^8^ and 456 R genes from *O*. *sativa*^22^ and performed a Pfam_Scan to search for domains in these genes. Fifty-five domains were obtained. Second, we used a hidden Markov Model (HMM) search tool^34^ to identify genes containing these 55 domains in the 13 species. Domains of identified genes were then extracted to check the domain compositions and orders in each gene. A subset of genes that contained the NBS domain were then chosen for further analysis. The N-terminal TIR domain was identified with the Pfam protein motif (PF01582; http://pfam.sanger.ac.uk/), while the N-terminal coiled-coil motif was identified by the tool NCOILS^39^.

### Tandem gene determination

We searched all tandem gene arrays across each genome for sequence homology and locations of genes in local regions. The determining rule for a TD array was set as: 1) each tandem gene array is composed of continuously distributed homologous genes with a sequence identity of: Blastp E-value < 1E-2), and 2) a tandem gene array should not be separated by insertion of more than five non-homologous genes.

### Synteny analysis of R genes

Pairwise genome analyses were performed to detect orthologous gene pairs. First, we used Blastp to identify homologous genes (E-values < 1E-20) between pairwise genomes. These homologous gene pairs were then submitted to check the homologous status of their flanking genes. Genes located in both flanking regions of the homologous gene pair were counted if they were the best hits of Blastp between the two species. Homologous gene pairs were considered as syntenic genes between the pairwise genomes if they had the highest ratio of homologous flanking genes.

### Alignment and phylogenetic tree analysis

Protein sequences were aligned using MUSCLE^40^ with default parameters. Then the aligned sequences were used to build a phylogenetic tree by MEGA using the Neighbor-Joining or Maximum-likelihood algorithms. Bootstrap tests were used to assign the confidence level of each branch of the tree.

### K_a_/K_s_ analysis

Protein sequences between pairs of homologous genes were aligned by MUSCLE^40^. Protein alignments were then translated into coding sequence alignments using an in-house Perl script. After that, *K*_a_ and *K*_s_ values were calculated based on the coding sequence alignments using the method of Nei and Gojobori as implemented in KaKs_calculator^41^. *K*_a_/*K*_s_ values of syntenic orthologs between pairs of the 13 species were then plotted as histograms. Meanwhile, the *K*_s_ loci were chosen from the aligned coding sequences and concatenated to build the phylogenetic tree.

### Variants calling and annotation

*S*. *italica* resequencing data were analyzed following the method described previously^35^. Low-quality reads were removed: (1) if one read contained >5% “N” bases; (2) if the average Phred-like score was <20; (3) if less than 40-bp remained after a test trim of the 3ʹ nucleotides with a Phred-like score <13; (4) if read-pairs were exact duplicates. Filtered reads were mapped to the reference genome of *S*. *italica* (version 1.0) using the “mem” algorithm of the Burrows-Wheeler Aligner (BWA)^42^. Variants were identified using Samtools^43^. Only loci with two alleles and Minor Allele Frequency (MAF) < = 0.05 were kept for further analysis.

For the information concerning gene prediction coordinates, variants were classified as being inter-genic, CDS, Intronic, or UTR. Variants in CDS were further separated into two types: those causing changes to coding proteins (non-synonymous SNPs and frame shift InDels) and synonymous SNPs and InDels without frame-shifts. Intronic variants were divided into splice site mutations (within 2-bp of splice site) and others.

### Genome diversity and selection

We used two measures to estimate the selection pressure on the genome of *S*. *italica* populations: π and Tajima’s D. The statistic π measures genomic diversity^37^, computing the average difference per locus over each pair of accessions; Tajima’s D evaluates selection pressure to detect the genomic regions under purifying or balancing selection^38^. Both measures were calculated for variants in each *S*. *italica* gene.

## Supplementary information

Supplementary Information

## References

[1] Dangl JL, Jones JD (2001). Plant pathogens and integrated defence responses to infection. Nature.

[2] McDowell JM, Woffenden BJ (2003). Plant disease resistance genes: recent insights and potential applications. Trends Biotechnol.

[3] Meyers BC, Kaushik S, Nandety RS (2005). Evolving disease resistance genes. Curr Opin Plant Biol.

[4] Flor H (1956). The Complementary Genic Systems in Flax and Flax Rust*. Advances in genetics.

[5] Flor H (1971). Current status of the gene-for-gene concept. Annual Review of Phytopathology.

[6] Hulbert SH (2001). Resistance gene complexes: evolution and utilization. Annu Rev Phytopathol.

[7] Kohler A (2008). Genome-wide identification of NBS resistance genes in Populus trichocarpa. Plant Molecular Biology.

[8] Meyers BC (2003). Genome-wide analysis of NBS-LRR-encoding genes in Arabidopsis. Plant Cell.

[9] Monosi B (2004). Full-genome analysis of resistance gene homologues in rice. Theor Appl Genet.

[10] Mun JH (2009). Genome-wide identification of NBS-encoding resistance genes in Brassica rapa. Mol Genet Genomics.

[11] Cannon SB (2002). Diversity, distribution, and ancient taxonomic relationships within the TIR and non-TIR NBS-LRR resistance gene subfamilies. J Mol Evol.

[12] Meyers BC (1999). Plant disease resistance genes encode members of an ancient and diverse protein family within the nucleotide-binding superfamily. Plant J.

[13] Pan Q, Wendel J, Fluhr R (2000). Divergent evolution of plant NBS-LRR resistance gene homologues in dicot and cereal genomes. J Mol Evol.

[14] Akita M, Valkonen JP (2002). A novel gene family in moss (Physcomitrella patens) shows sequence homology and a phylogenetic relationship with the TIR-NBS class of plant disease resistance genes. J Mol Evol.

[15] Meyers BC, Morgante M, Michelmore RW (2002). TIR-X and TIR-NBS proteins: two new families related to disease resistance TIR-NBS-LRR proteins encoded in Arabidopsis and other plant genomes. Plant J.

[16] Leister D (2004). Tandem and segmental gene duplication and recombination in the evolution of plant disease resistance gene. Trends Genet.

[17] Porter BW (2009). Genome-wide analysis of Carica papaya reveals a small NBS resistance gene family. Mol Genet Genomics.

[18] Ameline-Torregrosa C (2008). Identification and characterization of nucleotide-binding site-leucine-rich repeat genes in the model plant Medicago truncatula. Plant Physiol.

[19] Yang S (2008). Recent duplications dominate NBS-encoding gene expansion in two woody species. Mol Genet Genomics.

[20] Michelmore RW, Meyers BC (1998). Clusters of resistance genes in plants evolve by divergent selection and a birth-and-death process. Genome Res.

[21] Richly E, Kurth J, Leister D (2002). Mode of amplification and reorganization of resistance genes during recent Arabidopsis thaliana evolution. Mol Biol Evol.

[22] Zhou T (2004). Genome-wide identification of NBS genes in japonica rice reveals significant expansion of divergent non-TIR NBS-LRR genes. Mol Genet Genomics.

[23] Liu J (2007). Recent progress in elucidating the structure, function and evolution of disease resistance genes in plants. J Genet Genomics.

[24] Zhao B (2005). A maize resistance gene functions against bacterial streak disease in rice. Proc Natl Acad Sci USA.

[25] Lin F (2007). The blast resistance gene Pi37 encodes a nucleotide binding site leucine-rich repeat protein and is a member of a resistance gene cluster on rice chromosome 1. Genetics.

[26] Zeng X (2011). Characterization and fine mapping of the rice blast resistance gene Pia. Sci China Life Sci.

[27] Wu Y (2013). Fine mapping and identification of blast resistance gene Pi-hk1 in a broad-spectrum resistant japonica rice landrace. Phytopathology.

[28] Guo C (2016). Cloning of novel rice blast resistance genes from two rapidly evolving NBS-LRR gene families in rice. Plant Mol Biol.

[29] Yang S (2013). Rapidly evolving R genes in diverse grass species confer resistance to rice blast disease. Proc Natl Acad Sci USA.

[30] Zhao Y (2016). Bioinformatics Analysis of NBS-LRR Encoding Resistance Genes in Setaria italica. Biochem Genet.

[31] Ayliffe MA, Lagudah ES (2004). Molecular genetics of disease resistance in cereals. Ann Bot.

[32] Luo S (2012). Dynamic nucleotide-binding site and leucine-rich repeat-encoding genes in the grass family. Plant Physiol.

[33] Sonnhammer EL (1998). Pfam: multiple sequence alignments and HMM-profiles of protein domains. Nucleic Acids Res.

[34] Eddy SR (1998). Profile hidden Markov models. Bioinformatics.

[35] Jia G (2013). A haplotype map of genomic variations and genome-wide association studies of agronomic traits in foxtail millet (Setaria italica). Nat Genet.

[36] Bennetzen JL (2012). Reference genome sequence of the model plant Setaria. Nat Biotechnol.

[37] Xu X (2012). Resequencing 50 accessions of cultivated and wild rice yields markers for identifying agronomically important genes. Nat Biotechnol.

[38] Tajima F (1983). Evolutionary relationship of DNA sequences in finite populations. Genetics.

[39] Koretke KK (1999). Fold recognition using sequence and secondary structure information. Proteins.

[40] Edgar RC (2004). MUSCLE: multiple sequence alignment with high accuracy and high throughput. Nucleic Acids Res.

[41] Zhang Z (2006). KaKs_Calculator: calculating Ka and Ks through model selection and model averaging. Genomics Proteomics Bioinformatics.

[42] Li H, Durbin R (2009). Fast and accurate short read alignment with Burrows-Wheeler transform. Bioinformatics.

[43] Li H (2009). The Sequence Alignment/Map format and SAMtools. Bioinformatics.

