# Supplementary material for: Comparative Genomics Analysis in Grass Species Reveals Two Distinct Evolutionary Strategies Adopted by R Genes
Source: Sci Rep. 2019 Jul 24;9:10735. doi: 10.1038/s41598-019-47121-8 (PMC6656885; doi:10.1038/s41598-019-47121-8)
Supplement: Supplementary file 1 — Supplementary Information [file 41598_2019_47121_MOESM1_ESM.docx]

**Comparative Genomics Analysis in Grass Species Reveals Two Distinct Evolutionary Strategies Adopted by R Genes**

Yinan Zhang, Meijun Guo, Jie Shen, Xie Song, Shuqi Dong, Yinyuan Wen, Xiangyang Yuan, and Pingyi Guo*

Agronomy College, Shanxi Agricultural University, Taigu, 030801, China

Author for correspondence: Pingyi Guo (E-mail: pyguo126@126.com)

**Supplementary Table S1.** The ID of 55 domains identified from the two R gene-sets of *O. sativa* and *A. thaliana*.

| **Index** | **Domain ID** |
| --- | --- |
| 1 | PF07723.12 |
| 2 | PF12799.6 |
| 3 | PF13306.5 |
| 4 | PF13516.5 |
| 5 | PF14580.5 |
| 6 | PF00931.21 |
| 7 | PF05627.10 |
| 8 | PF13855.5 |
| 9 | PF00646.32 |
| 10 | PF13713.5 |
| 11 | PF08381.10 |
| 13 | PF07725.11 |
| 14 | PF00560.32 |
| 15 | PF03018.13 |
| 16 | PF08263.11 |
| 17 | PF01061.23 |
| 18 | PF08370.10 |
| 19 | PF00005.26 |
| 20 | PF07714.16 |
| 21 | PF00069.24 |
| 22 | PF06479.11 |
| 23 | PF14510.5 |
| 24 | PF12819.6 |
| 25 | PF08387.9 |
| 26 | PF00664.22 |
| 27 | PF04632.11 |
| 28 | PF01419.16 |
| 29 | PF01566.17 |
| 30 | PF08627.9 |
| 31 | PF12937.6 |
| 32 | PF03106.14 |
| 33 | PF00085.19 |
| 34 | PF03479.14 |
| 35 | PF05659.10 |
| 36 | PF06963.11 |
| 37 | PF00498.25 |
| 38 | PF00569.16 |
| 39 | PF13764.5 |
| 40 | PF00240.22 |
| 41 | PF13676.5 |
| 42 | PF13966.5 |
| 43 | PF07228.11 |
| 44 | PF02362.20 |
| 45 | PF06507.12 |
| 46 | PF02309.15 |
| 47 | PF02892.14 |
| 48 | PF05678.13 |
| 49 | PF02535.21 |
| 50 | PF14291.5 |
| 51 | PF00903.24 |
| 52 | PF06838.10 |
| 53 | PF15699.4 |
| 54 | PF12740.6 |
| 55 | PF02537.14 |

**Supplementary Table S2.** The number of subgroups of NBS R genes in the 13 studied species.

|  | **Si** | **Sv** | **Pv** | **Ph** | **Sb** | **Zm** | **Ot** | **Ta** | **Bs** | **Bd** | **Os** | **Ma** | **At** | **Sum** | **TD** |
| --- | --- | --- | --- | --- | --- | --- | --- | --- | --- | --- | --- | --- | --- | --- | --- |
| **CC-NBS-LRR** | 159 | 120 | 238 | 95 | 118 | 46 | 2 | 666 | 99 | 126 | 175 | 43 | 21 | 1451 | 728 |
| **CC-NBS** | 41 | 47 | 196 | 48 | 33 | 23 | 13 | 435 | 42 | 40 | 68 | 13 | 9 | 799 | 285 |
| **CC-NBS-NBS** | 12 |  | 16 | 10 | 7 | 6 | 12 | 34 | 10 | 12 | 11 | 6 | 6 | 138 | 39 |
| **CC-NBS-NBS-LRR** | 14 | 9 | 17 | 9 | 6 | 3 |  | 39 | 10 | 15 | 9 | 1 | 10 | 106 | 68 |
| **CC-NBS-LRR-LRR** | 3 | 4 | 2 | 1 | 2 |  |  | 2 | 5 |  | 4 | 1 | 1 | 26 | 12 |
| **CC-NBS-LRR-NBS-LRR** |  | 9 | 2 |  | 1 | 1 |  | 7 | 1 | 1 | 2 | 2 |  | 21 | 5 |
| **CC-LRR-NBS-LRR** | 1 | 1 |  |  | 2 |  |  | 5 |  |  | 2 |  | 2 | 10 | 3 |
| **CC-NBS-NBS-NBS** |  | 1 |  |  |  | 1 | 1 |  |  | 1 | 1 |  |  | 5 | 3 |
| **CC-NBS-LRR-NBS** |  |  |  |  |  |  |  |  |  |  | 1 | 1 | 2 | 4 | 4 |
| **CC-NBS-NBS-NBS-LRR** |  |  |  |  |  |  |  | 3 | 1 | 3 |  |  |  | 4 | 2 |
| **CC-LRR-NBS-NBS-LRR** |  |  |  |  |  |  |  | 1 |  |  |  |  | 2 | 3 | 1 |
| **CC-NBS-NBS-LRR-LRR** |  |  |  |  |  |  |  | 1 |  | 1 |  |  | 1 | 3 |  |
| **CC-NBS-NBS-NBS-NBS** |  |  |  |  |  | 1 |  |  |  |  |  |  | 1 | 2 | 1 |
| **CC-LRR-NBS** |  |  | 1 |  |  |  |  |  |  |  |  |  |  | 1 | 1 |
| **CC-NBS-NBS-LRR-NBS** |  |  |  |  |  |  |  |  |  |  |  | 1 |  | 1 | 1 |
| **CC-NBS-NBS-LRR-NBS-LRR** |  |  |  |  |  |  |  | 1 |  |  |  |  |  |  |  |
| **NBS-LRR** | 156 | 122 | 334 | 123 | 146 | 37 | 5 | 792 | 83 | 111 | 163 | 36 | 16 | 1704 | 757 |
| **NBS** | 88 | 90 | 336 | 76 | 56 | 66 | 38 | 541 | 70 | 84 | 99 | 45 | 26 | 1601 | 457 |
| **NBS-NBS** | 45 | 46 | 87 | 45 | 40 | 31 | 21 | 181 | 45 | 48 | 32 | 28 | 23 | 579 | 148 |
| **NBS-NBS-LRR** | 10 | 7 | 24 | 6 | 6 | 7 |  | 22 | 3 | 8 | 9 | 3 | 3 | 89 | 43 |
| **NBS-LRR-LRR** | 2 | 4 | 3 | 1 |  |  |  | 2 | 1 | 3 | 4 |  |  | 22 | 10 |
| **NBS-NBS-NBS** |  | 1 |  |  | 3 | 1 | 4 |  | 3 | 4 |  | 1 | 1 | 18 | 3 |
| **NBS-LRR-NBS-LRR** |  |  | 3 | 1 | 1 | 1 |  | 1 |  | 1 | 1 | 2 |  | 10 | 7 |
| **NBS-LRR-NBS** |  | 1 | 3 |  |  | 1 |  | 1 |  | 1 | 1 |  |  | 8 | 5 |
| **NBS-NBS-LRR-LRR** |  |  |  |  |  |  |  | 2 | 1 |  |  |  |  | 2 | 1 |
| **NBS-NBS-LRR-NBS-LRR** |  |  |  | 1 |  |  |  |  |  |  |  |  |  | 1 | 1 |
| **NBS-NBS-LRR-NBS-NBS** |  |  |  |  |  | 1 |  |  |  |  |  |  |  | 1 | 1 |
| **NBS-NBS-NBS-LRR** |  |  | 1 |  |  |  |  |  |  |  |  |  |  | 1 |  |
| **LRR-NBS-LRR** |  |  | 1 | 1 | 1 | 1 |  | 2 |  | 2 | 1 | 2 | 1 | 11 | 9 |
| **LRR-NBS-LRR-NBS-LRR** |  |  |  |  |  | 1 |  |  |  |  |  | 1 |  | 2 | 1 |
| **LRR-NBS** |  |  | 1 |  |  |  |  |  |  |  |  |  |  | 1 | 1 |
| **LRR-NBS-NBS** |  |  |  |  |  |  |  | 1 |  |  |  |  |  |  |  |
| **LRR-NBS-NBS-LRR** | 1 |  |  |  |  |  |  |  |  |  |  |  |  | 1 |  |
| ***TIR-NBS-LRR*** |  |  |  |  |  |  |  |  |  |  |  |  | **66** | 66 | 47 |
| ***TIR-NBS*** | 2 | 2 | 1 | 1 | 1 | 2 |  | 2 | 3 | 3 | 4 | 3 | **11** | 35 | 9 |
| ***CC-TIR-NBS-LRR*** |  |  |  |  |  |  |  | 7 |  |  |  |  | **9** | 9 | 8 |
| ***TIR-NBS-TIR*** | 1 |  |  | 1 |  |  |  |  |  |  |  | 1 | **2** | 5 | 2 |
| ***TIR-TIR-NBS*** |  |  |  | 1 | 1 |  | 1 | 2 |  |  |  |  |  | 5 |  |
| ***TIR-NBS-LRR-LRR*** |  |  |  |  |  |  |  |  |  |  |  |  | **2** | 2 | 1 |
| ***TIR-NBS-NBS*** |  |  | 1 |  |  |  |  |  |  |  |  |  | **1** | 2 | 1 |
| ***TIR-NBS-TIR-LRR*** |  |  |  |  |  |  |  |  |  |  |  |  | **2** | 2 | 2 |
| ***TIR-NBS-TIR-NBS-LRR*** | |  |  |  |  |  |  |  |  |  |  |  | **2** | 2 | 1 |
| ***CC-TIR-NBS*** |  |  |  |  |  |  |  |  |  |  |  |  | **2** | 2 | 1 |
| ***TIR-NBS-LRR-NBS*** |  |  |  |  |  |  |  |  |  |  |  |  | **1** | 1 | 1 |
| ***TIR-NBS-LRR-TIR*** |  |  |  |  |  |  |  |  |  |  |  |  | **1** | 1 | 1 |
| ***TIR-NBS-NBS-LRR*** |  |  |  |  |  |  |  |  |  |  |  |  | **1** | 1 | 1 |
| ***TIR-TIR-NBS-TIR*** |  | 1 |  |  |  |  |  |  |  |  |  |  |  | 1 |  |
| ***TIR-TIR-NBS-TIR-NBS-LRR*** | |  |  |  |  |  |  |  |  |  |  |  | **1** | 1 | 1 |
| ***CC-TIR-NBS-TIR*** |  |  |  |  |  |  |  |  |  |  |  |  | **1** | 1 | 1 |
| ***CC-TIR-NBS-TIR-LRR*** |  |  |  |  | 1 |  |  |  |  |  |  |  |  | 1 |  |
| ***CC-TIR-TIR-NBS-LRR-TIR*** | |  |  |  |  |  |  |  |  |  |  |  | **1** | 1 | 1 |
| ***NBS-LRR-TIR*** |  |  |  |  |  |  |  |  |  |  |  |  | **1** | 1 | 1 |
| ***NBS-TIR*** |  |  |  |  |  |  |  | 1 |  |  |  |  |  | 1 |  |
| **Total** | 535 | 465 | 1267 | 420 | 425 | 230 | 97 | 2747 | 377 | 464 | 587 | 190 | 229 | 6765 | 2676 |

*Note: Si: Setaria italica; Sv: Setaria viridis; Pv: Panicum virgatum; Ph: Panicum hallii; Sb: Sorghum bicolor; Zm: Zea mays; Ot: Oropetium thomaeum; Ta: Triticum aestivum; Bs: Brachypodium stacei; Bd: Brachypodium distachyon; Os: Oryza sativa; Ma: Musa acuminate; At: Arabidopsis thaliana.*

**Supplementary Table S3.** The number of syntenic gene pairs between each two of the 13 species studied.

|  | ***S. italica*** | *S. viridis* | *P. virgatum* | *P. hallii* | *S. bicolor* | *Z. mays* | *O. thomaeum* | *T. aestivum* | *B. stacei* | *B. distachyon* | *O. sativa* | *M. acuminate* | *A. thaliana* |
| --- | --- | --- | --- | --- | --- | --- | --- | --- | --- | --- | --- | --- | --- |
| ***S. italica*** | - | 26328 | 40079 | 20468 | 19443 | 22062 | 19477 | 42393 | 16973 | 17345 | 17851 | 1840 | 130 |
| *S. viridis* | - | - | 39560 | 20343 | 19318 | 21849 | 19372 | 41905 | 16885 | 17141 | 17682 | 1736 | 54 |
| *P. virgatum* | - | - | - | 41803 | 36616 | 19910 | 27773 | 27075 | 30662 | 30789 | 32297 | 258 | 200 |
| *P. hallii* | - | - | - | - | 18720 | 21129 | 18504 | 40525 | 16117 | 16248 | 16811 | 1635 | 81 |
| *S. bicolor* | - | - | - | - | - | 23694 | 18634 | 41130 | 16378 | 16569 | 17017 | 1737 | 103 |
| *Z. mays* | - | - | - | - | - | - | 18082 | 24123 | 19462 | 19610 | 19979 | 325 | 2 |
| *O. thomaeum* | - | - | - | - | - | - | - | 34477 | 16269 | 16352 | 16990 | 945 | 119 |
| *T. aestivum* | - | - | - | - | - | - | - | - | 43097 | 43682 | 39574 | 34 | 271 |
| *B. stacei* | - | - | - | - | - | - | - |  | - | 21823 | 16434 | 1583 | 63 |
| *B. distachyon* | - | - | - | - | - | - | - |  | - | - | 16847 | 1482 | 129 |
| *O. sativa* | - | - | - | - | - | - | - |  | - | - | - | 1651 | 145 |
| *M. acuminate* | - | - | - | - | - | - | - |  | - | - | - | - | 29 |
| *A. thaliana* | - | - | - | - | - | - | - |  | - | - | - | - | - |

**Supplementary Table S4.** The number of species-specific R locus determined by non-synteny relationships among species.

| **Species** | **#non-synteny R locus** |
| --- | --- |
| *S. italica* | 30 |
| *S. viridis* | 22 |
| *P. hallii* | 61 |
| *S. bicolor* | 37 |
| *Z. mays* | 41 |
| *O. thomaeum* | 17 |
| *B. stacei* | 24 |
| *B. distachyon* | 51 |
| *O. sativa* | 143 |

**Supplementary Table S5.** The number of SNPs located at different genomic regions in relation to genes.

| **Coding region** | | **Intronic** | | **UTR** | | **Intergenic** | **Total** |
| --- | --- | --- | --- | --- | --- | --- | --- |
| **Non-Syn** | **Syn** | **Splice** | **Intron** | **5UTR** | **3UTR** |  |  |
| 70,229 | 54,600 | 442 | 178,811 | 214,487 | 185,867 | 1,451,151 | 2,156,941 |

*UTR for untranslated region; Syn for synonymous.*

**Supplementary Table S6.** The number of InDels located at different genomic regions in relation to genes.

| **Coding region** | | **Intronic** | | **UTR** | | **Intergenic** | **Total** |
| --- | --- | --- | --- | --- | --- | --- | --- |
| **Frame-shift** | **Non-frameshift** | **Splice** | **Intron** | **5UTR** | **3UTR** |  |  |
| 3,733 | 4,518 | 374 | 23,839 | 31,581 | 25,759 | 100,430 | 189,440 |

*Frame-shift: InDel variation causing frameshift of coding sequences.*

**Supplementary Table S7.** The syntenic orthologous genes of *Z. mays* bacterial streak disease resistance gene *Rxo1* in the other 11 grass species.

| **Species** | **Rxo1 synteny**  **orthologs** | **Identity %** | **Coverge %** | **E-value** | **Tandem array** |
| --- | --- | --- | --- | --- | --- |
| *O. sativa* | / | / | / | / | / |
| *B. distachyon* | *Bradi3g14033.3* | 27.17 | 57.80 | 1.19E-52 | / |
| *B. stacei* | *Brast03G044700.1* | 26.55 |  | 9.56E-55 | / |
| *T. aestivum* | / | / | / | / | / |
| *O. thomaeum* | / | / | / | / | / |
| *Z. mays* | *Zm00008a023587_P01* (***Rxo1***) | - | - | - | Zm00008a023587_P01;  Zm00008a023588_P01 |
| *S. bicolor* | *Sobic.007G027100.1* | 27.53 | 99.02 | 4.28E-76 | / |
| *P. hallii* | *Pahal.F00026.1* | 27.61 | 35.55 | 1.28E-24 | / |
| *P. virgatum* | / | / | / | / | / |
| *S. viridis* | *Sevir.6G071100.1* | 27.70 | 37.40 | 2.47E-26 | / |
| *S. italica* | *Seita.6G072000.1* | 27.66 | 48.09 | 1.28E-29 | / |


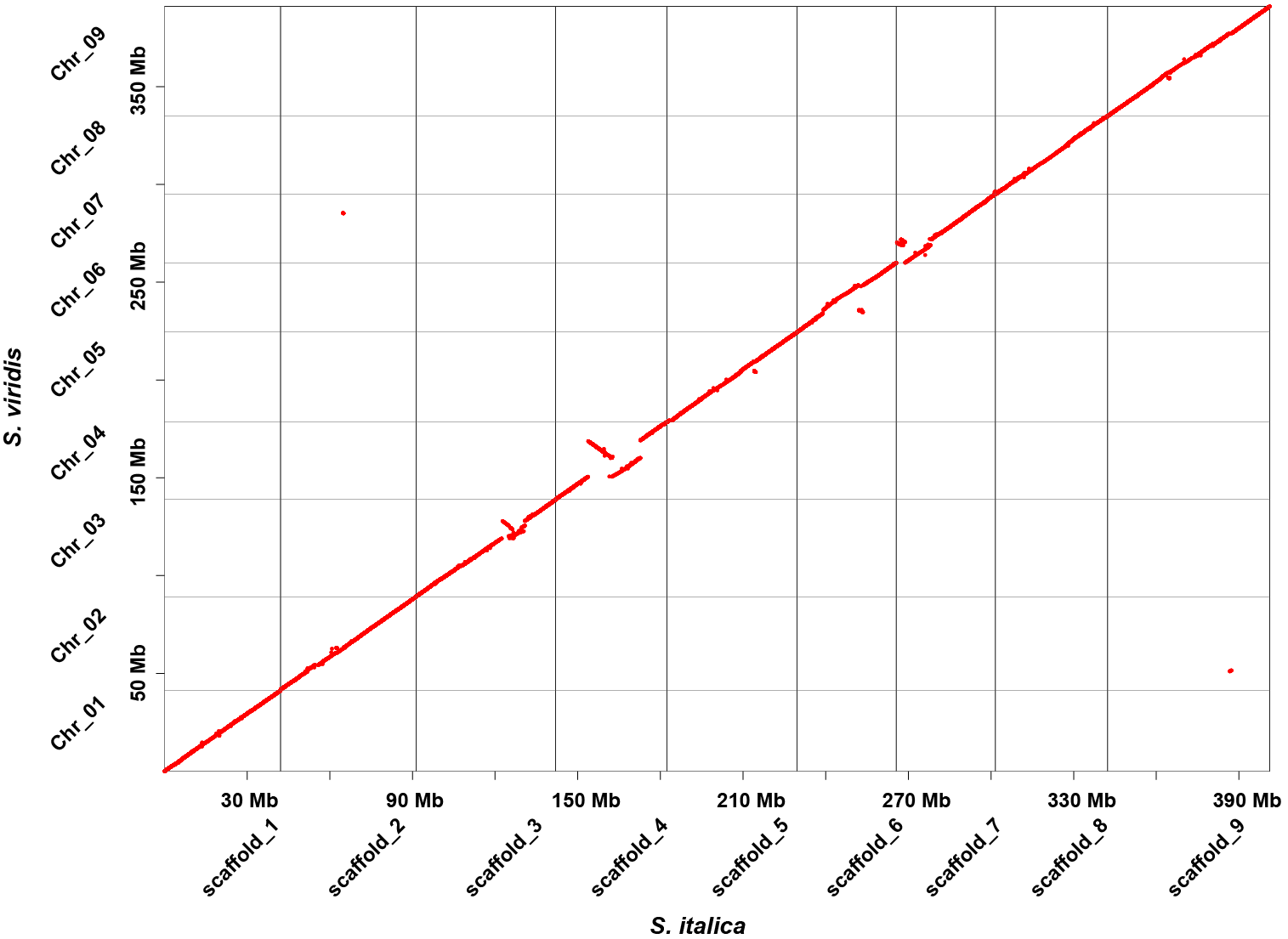


**Supplementary Figure S1.** Dotplot of syntenic gene pairs between *S. italica* and *S. viridis*.


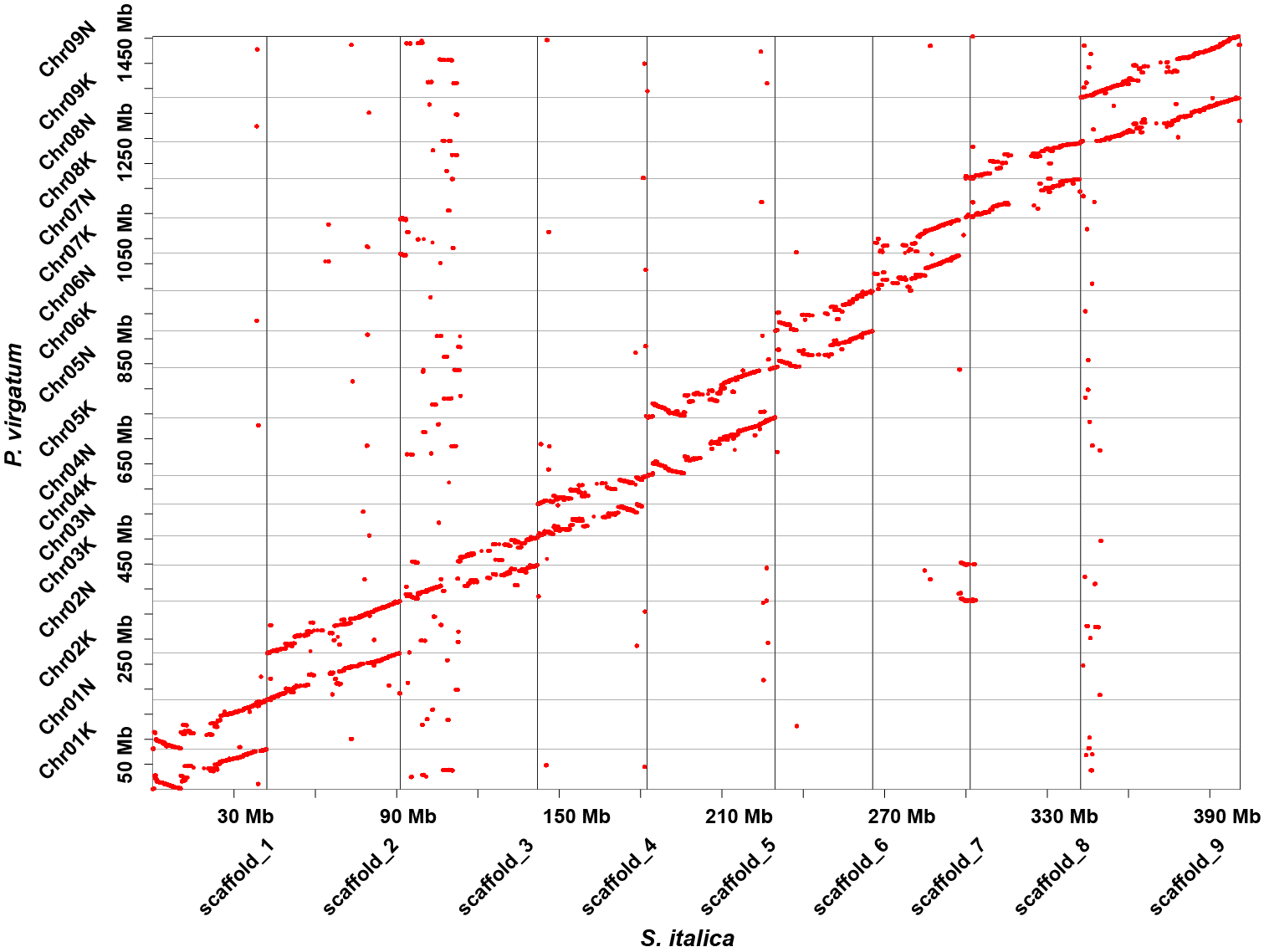


**Supplementary Figure S2.** Dotplot of syntenic gene pairs between *S. italica* and *P. virgatum*.


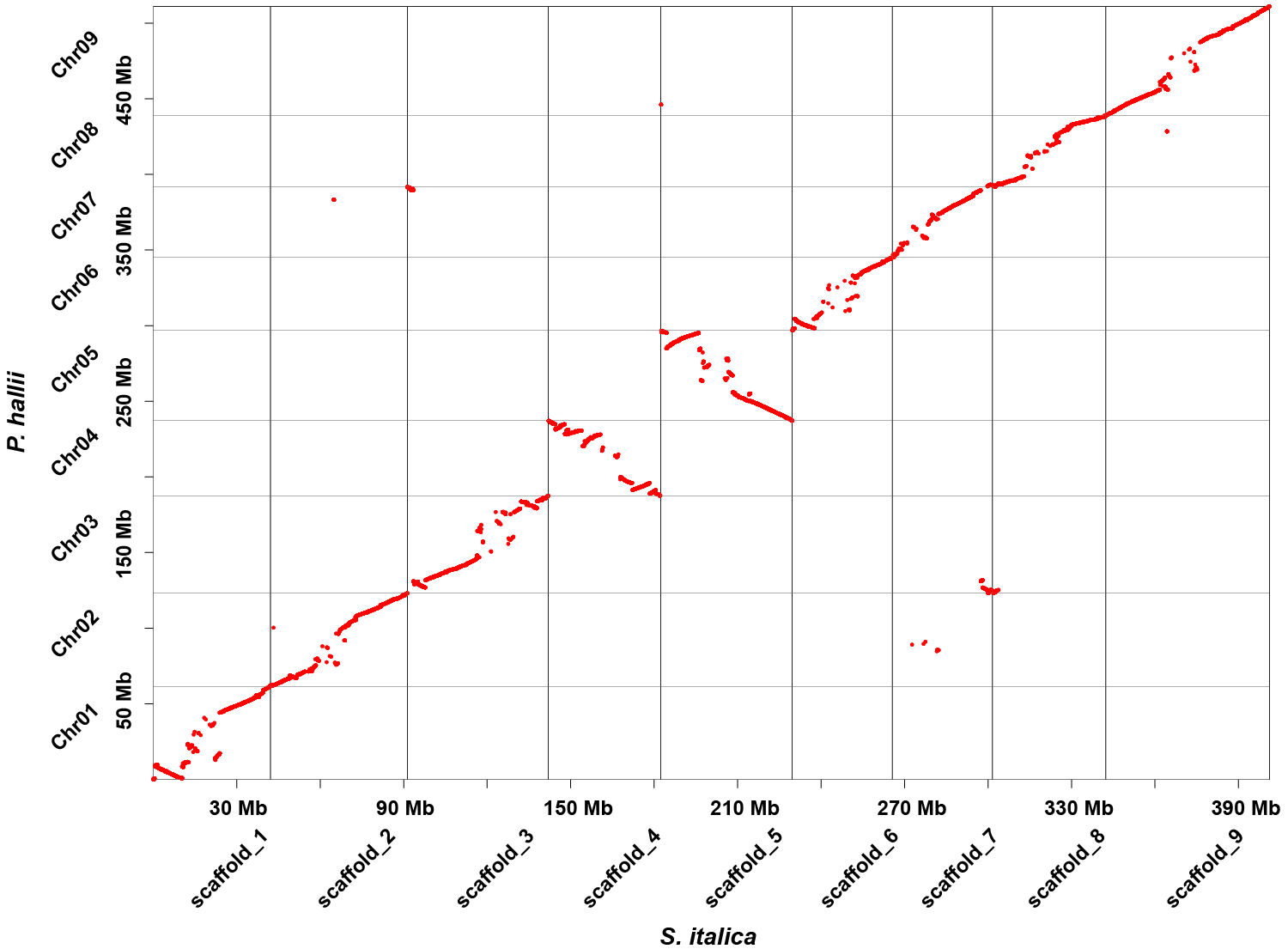


**Supplementary Figure S3.** Dotplot of syntenic gene pairs between *S. italica* and *P. hallii*.


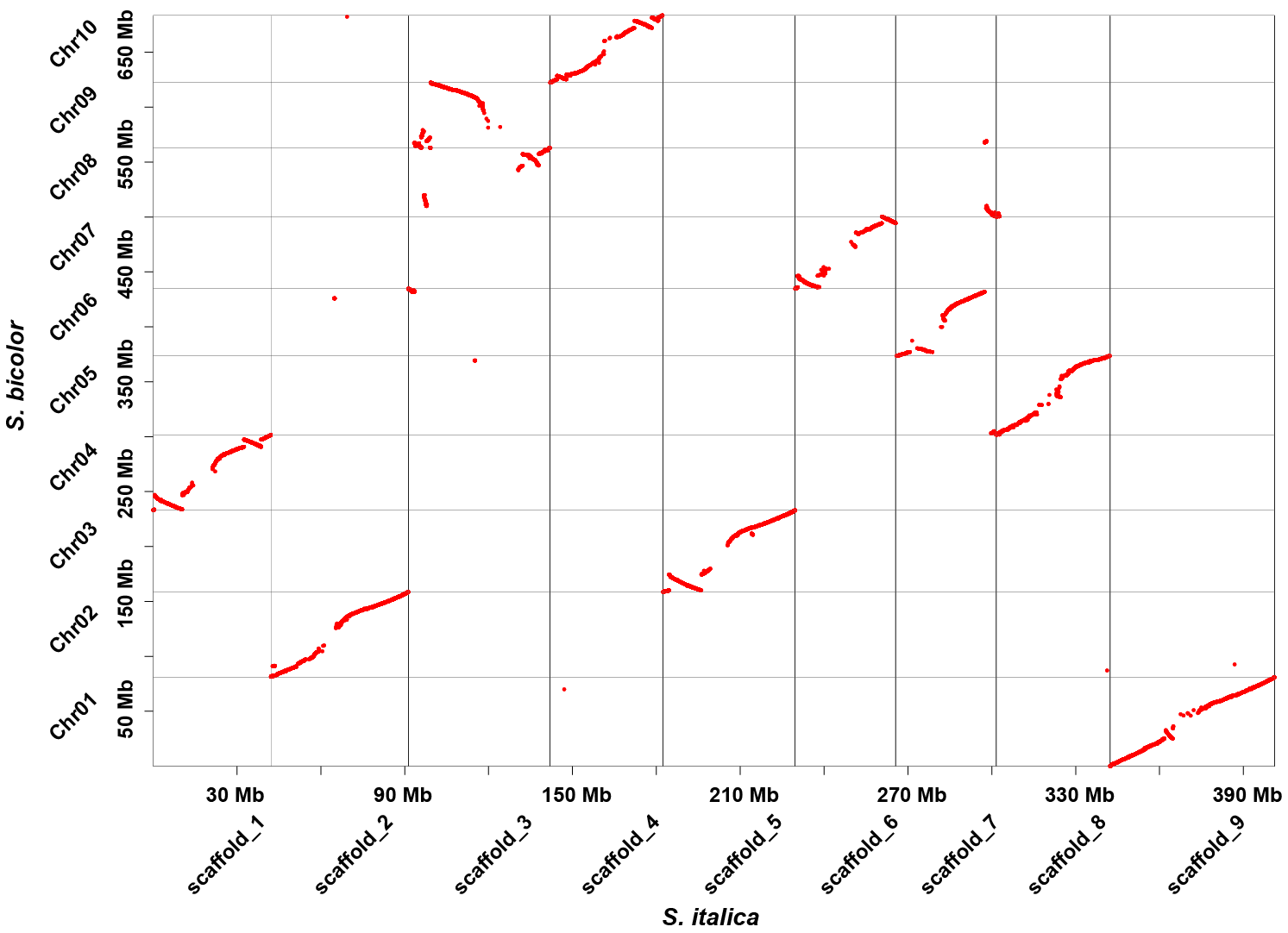


**Supplementary Figure S4.** Dotplot of syntenic gene pairs between *S. italica* and *S. bicolor*.


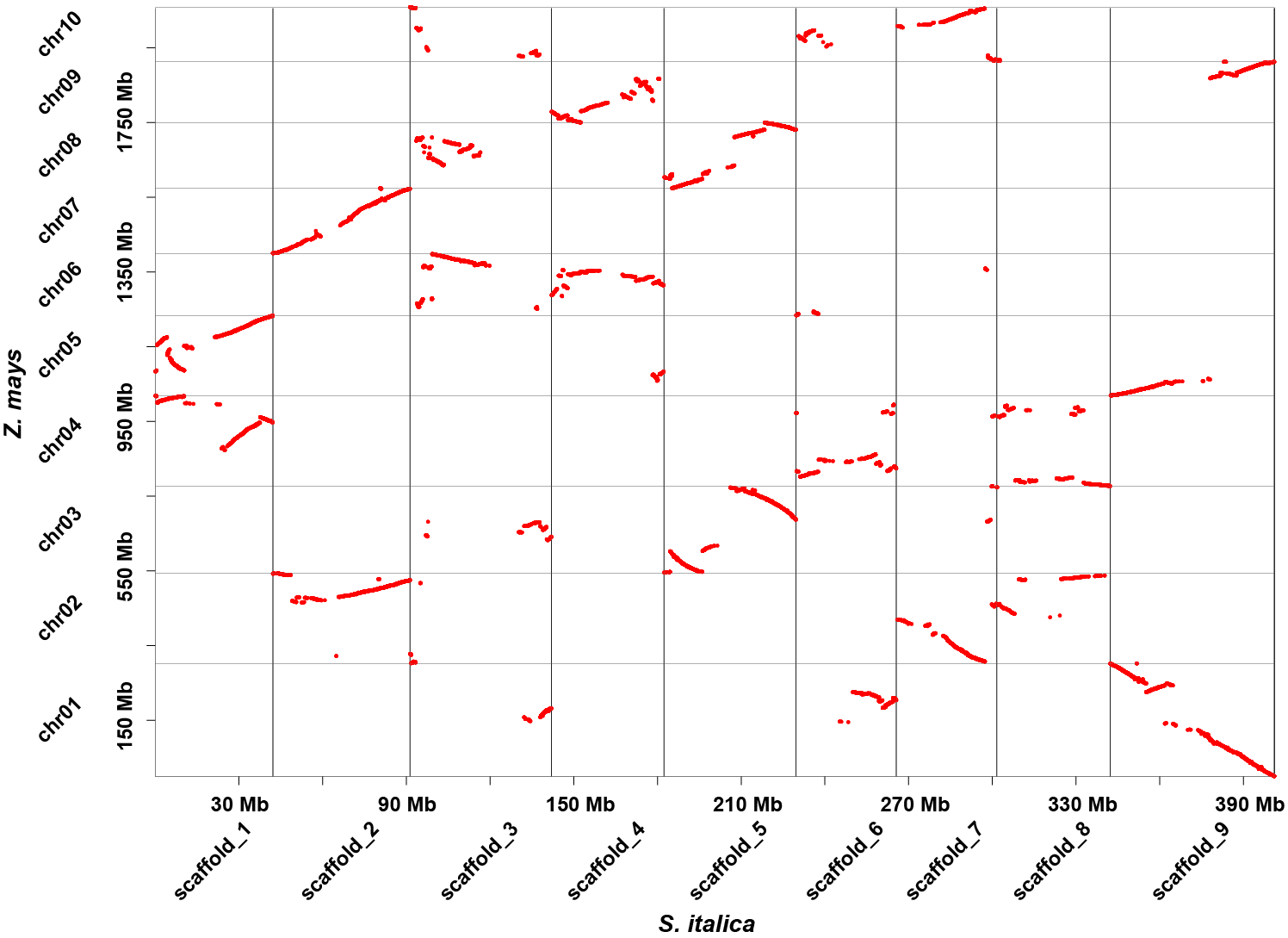


**Supplementary Figure S5.** Dotplot of syntenic gene pairs between *S. italica* and *Z. mays*.


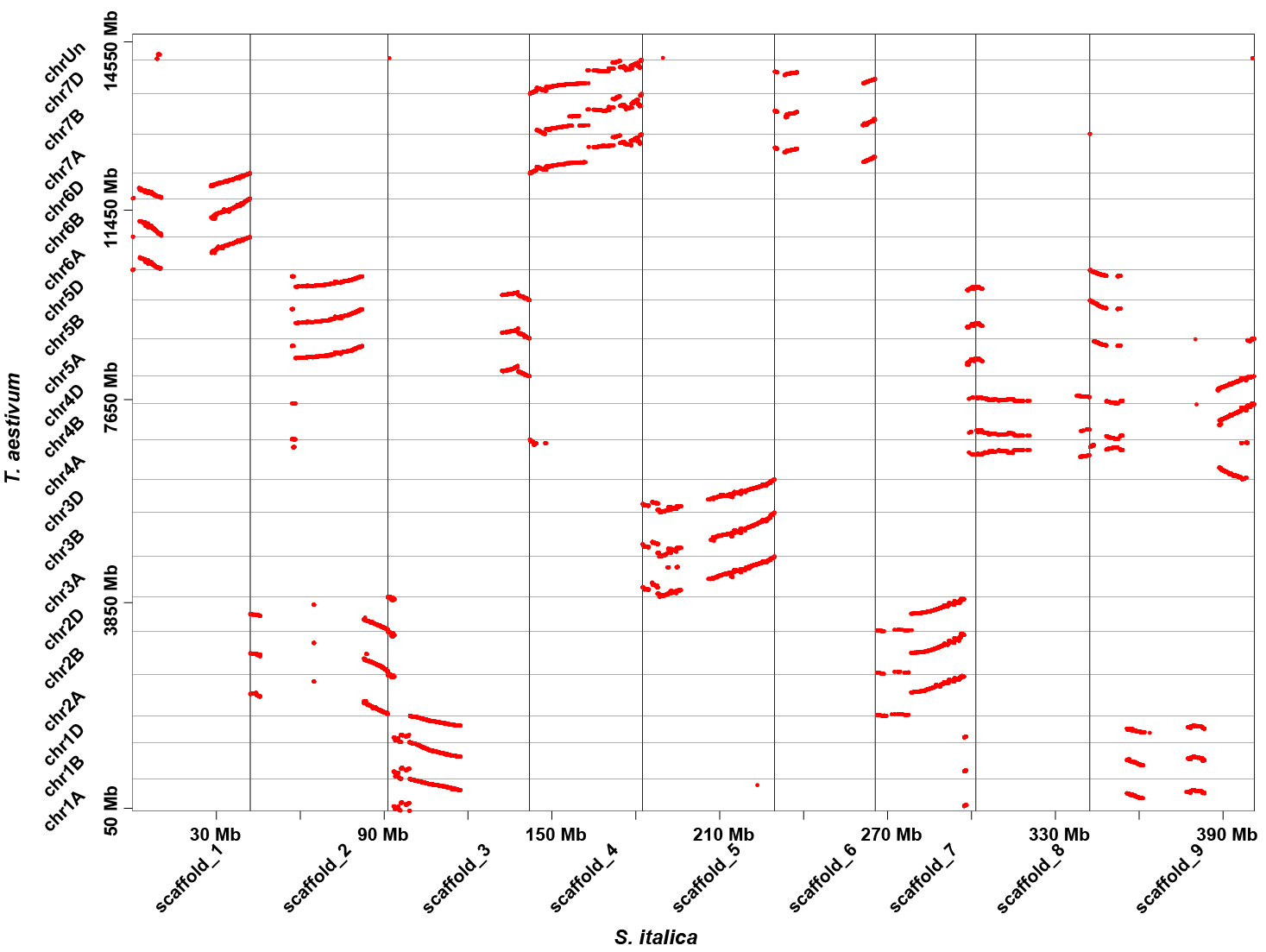


**Supplementary Figure S6.** Dotplot of syntenic gene pairs between *S. italica* and *T. aestivum*.


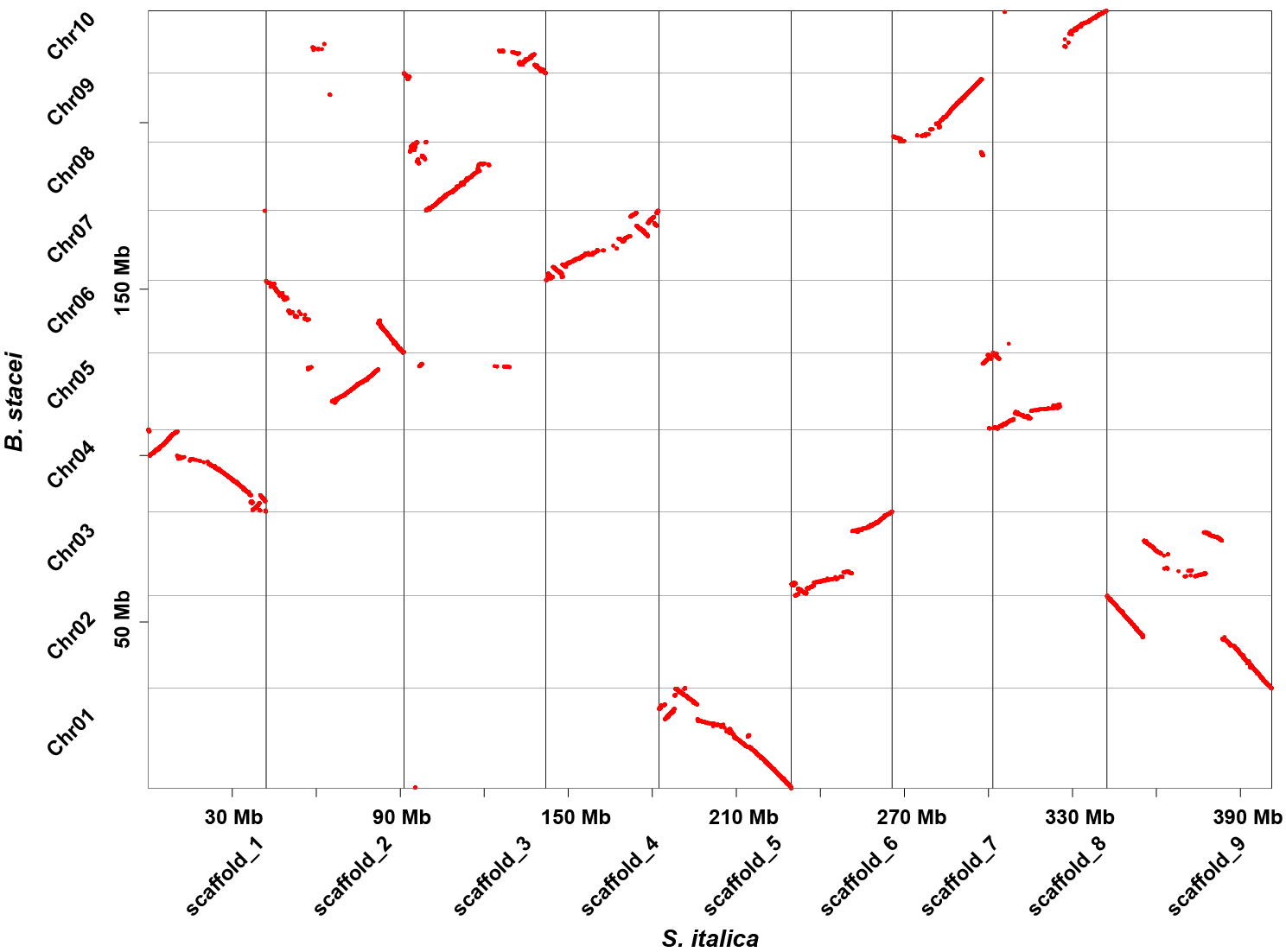


**Supplementary Figure S7.** Dotplot of syntenic gene pairs between *S. italica* and *B. stacei*.


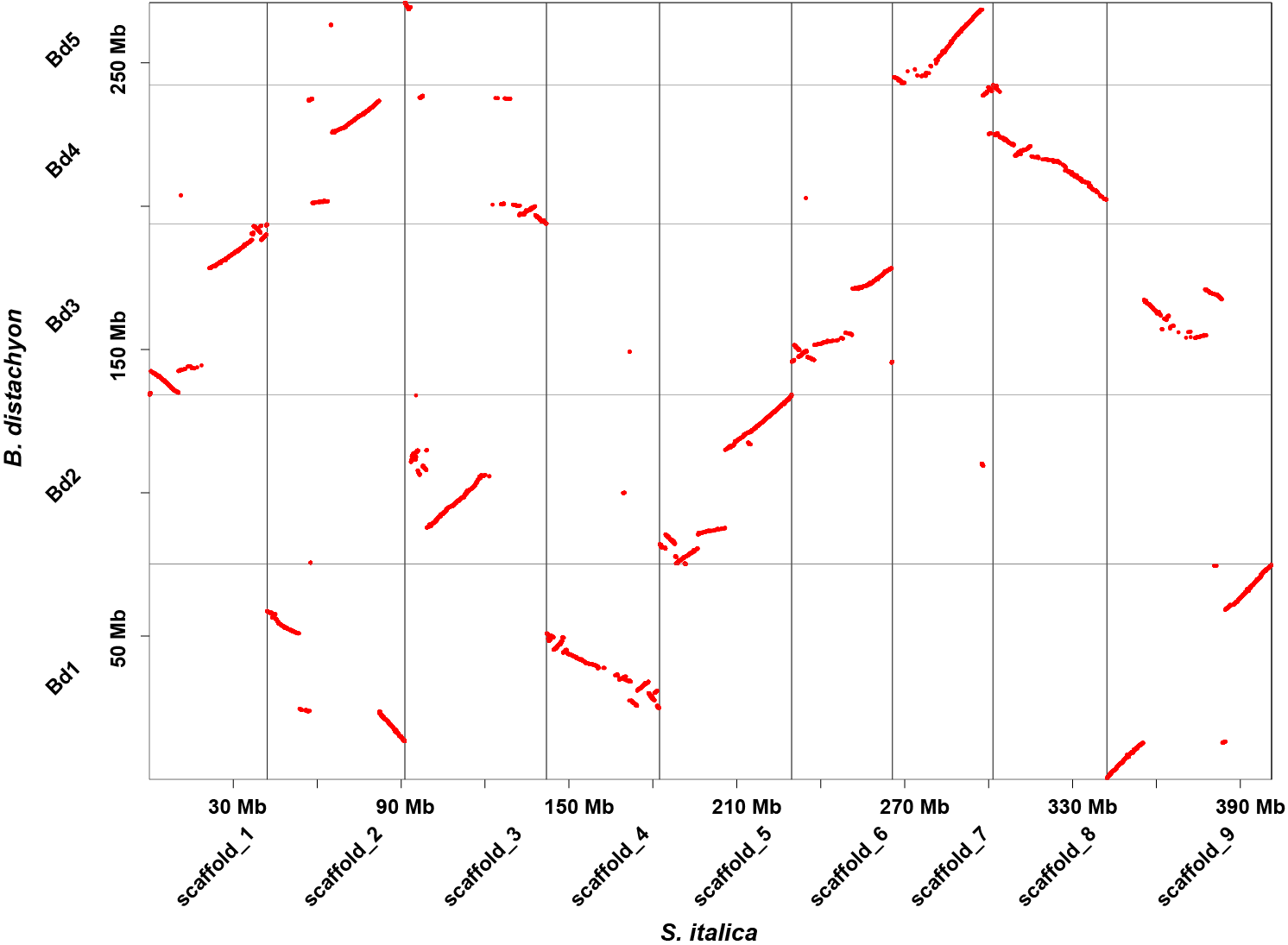


**Supplementary Figure S8.** Dotplot of syntenic gene pairs between *S. italica* and *B. distachyon*.


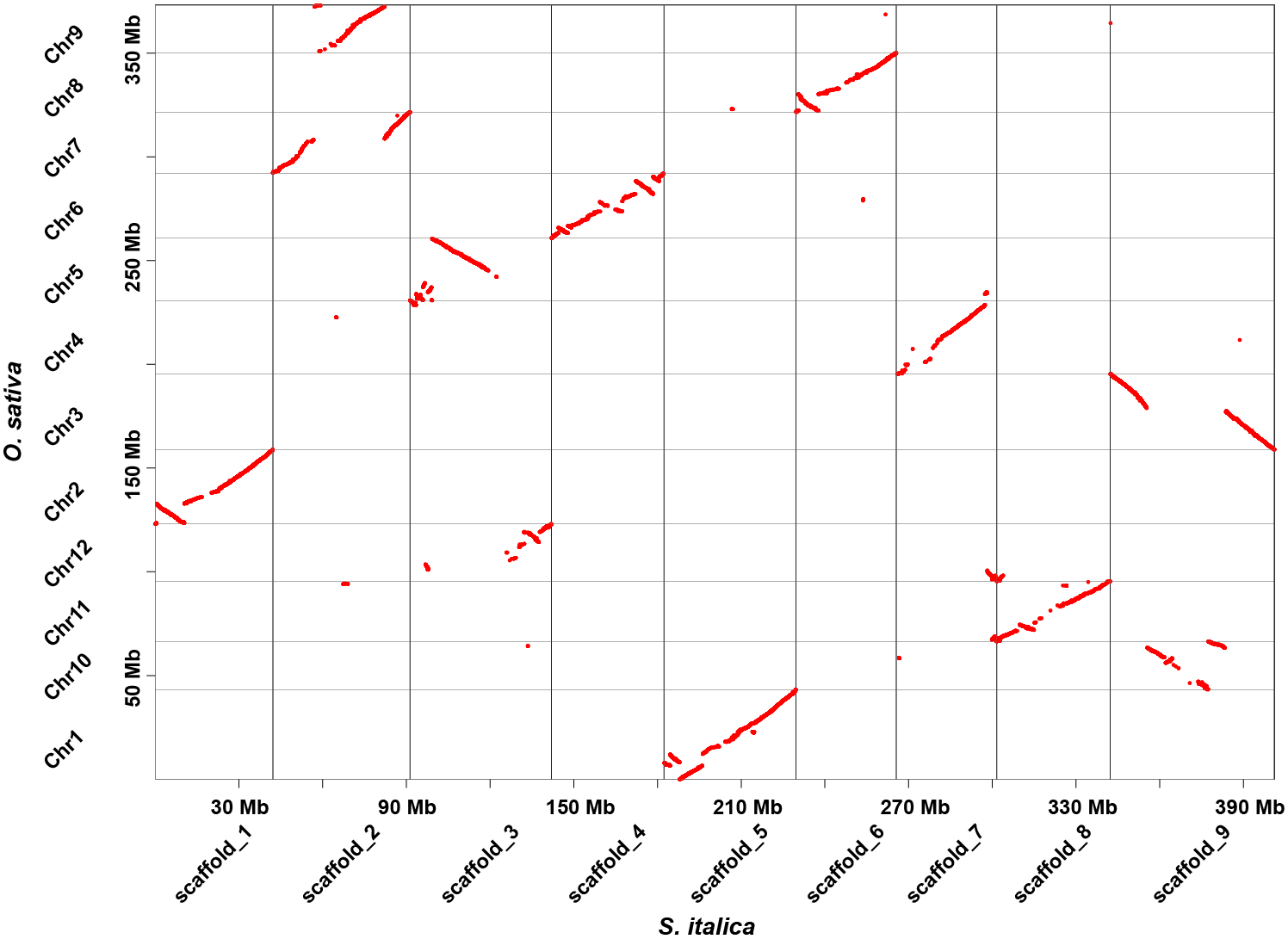


**Supplementary Figure S9.** Dotplot of syntenic gene pairs between *S. italica* and *O. sativa*.


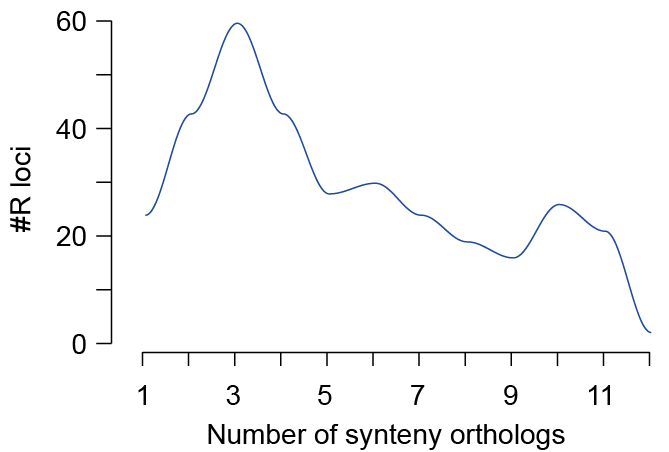


**Supplementary Figure S10.** The distribution of the number of syntenic R orthologous genes among the 13 species studied.


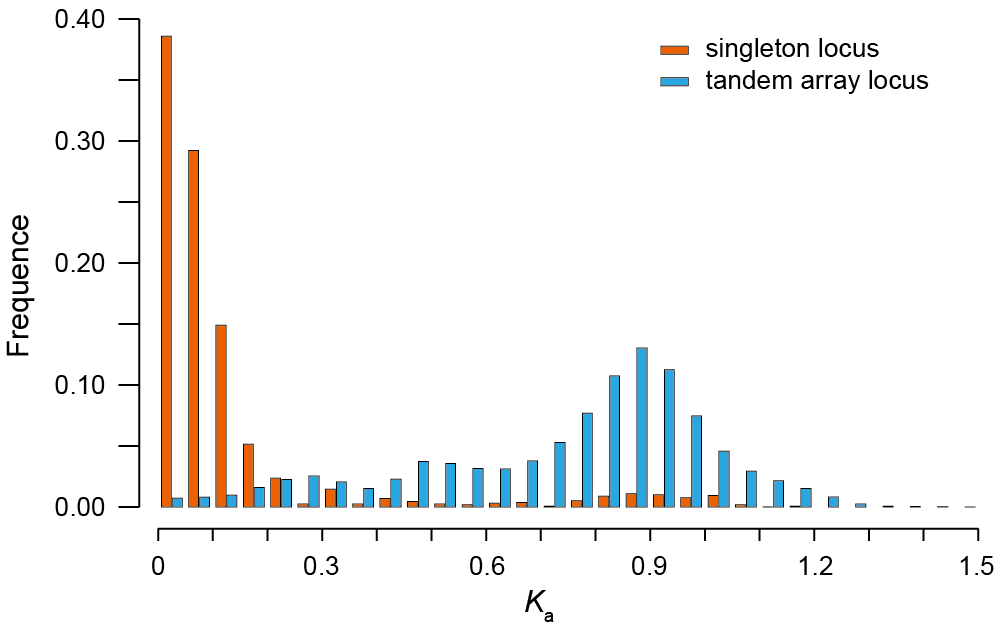


**Supplementary Figure S11.** The frequency distribution of *K*_a_ values between syntenic R gene pairs from R singleton (orange) and R TD (light-blue) loci.


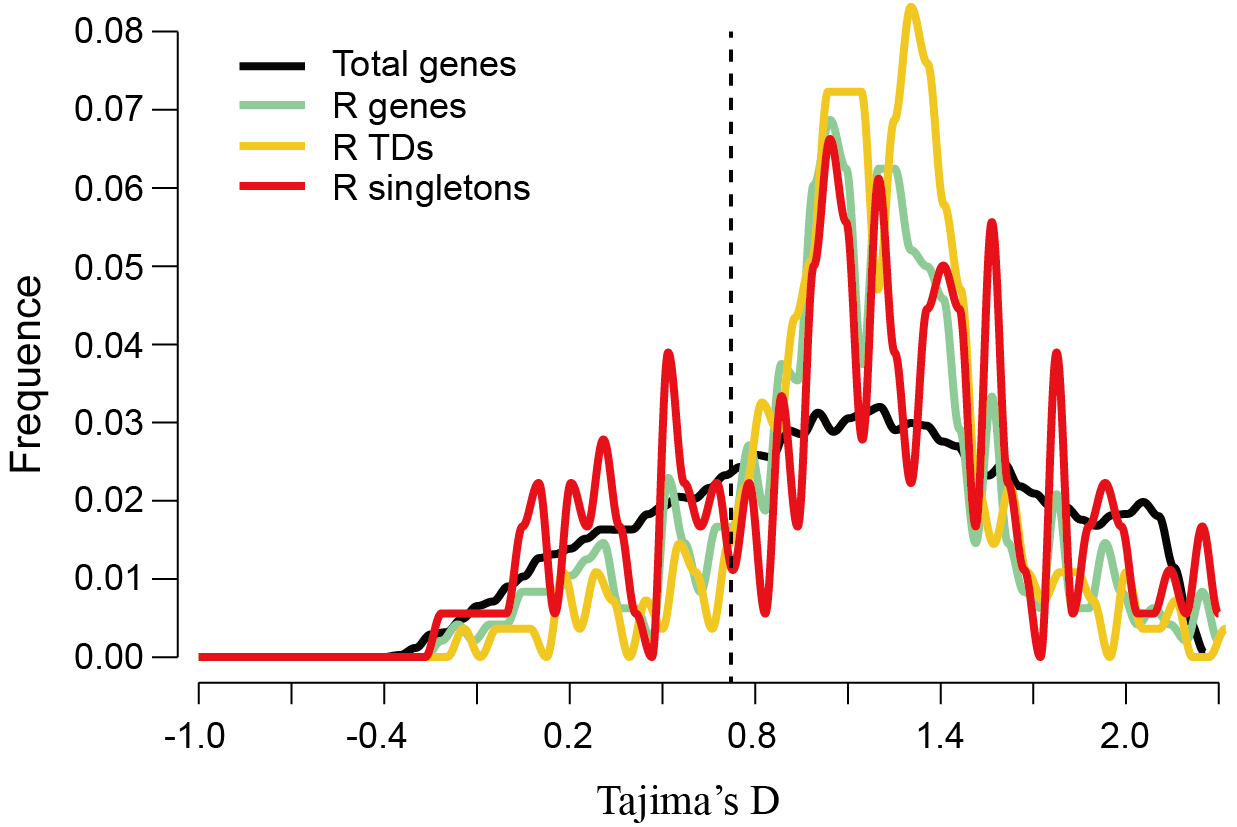


**Supplementary Figure S12.** The distribution of Tajima’s D values of total genes and different sets of R genes in population of *S. italica*.
